# Supplementary material for: Development and Assessment of SNP Genotyping Arrays for Citrus and Its Close Relatives
Source: Plants (Basel). 2024 Feb 29;13(5):691. doi: 10.3390/plants13050691 (PMC10935022; doi:10.3390/plants13050691)

Supplementary Material

Figure S1 Venn diagram of 1.3M autosomal variants for the Axiom® Citrus HD Genotyping Array. Venn diagram shows the number of polymorphic variants, after variant filtering part 1, contributed by each group represented in the discovery panel and used on the Axiom® Citrus HD Genotyping Array.

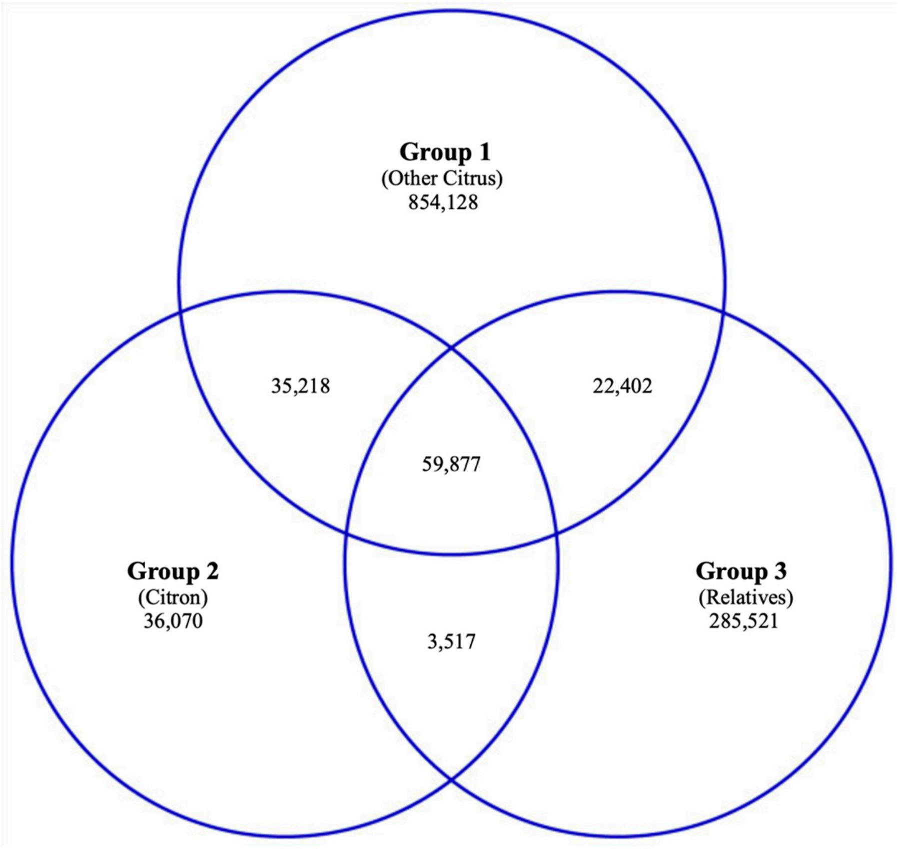

Figure S2. Distribution of SNPs represented on Axiom® Citrus HD Genotyping Array on each of nine chromosomes. SNP distribution is plotted against Phytozome *C. clementina v1.0* reference genome on chromosome 1-9 (a-i) with 500kb window size and 5kb step size

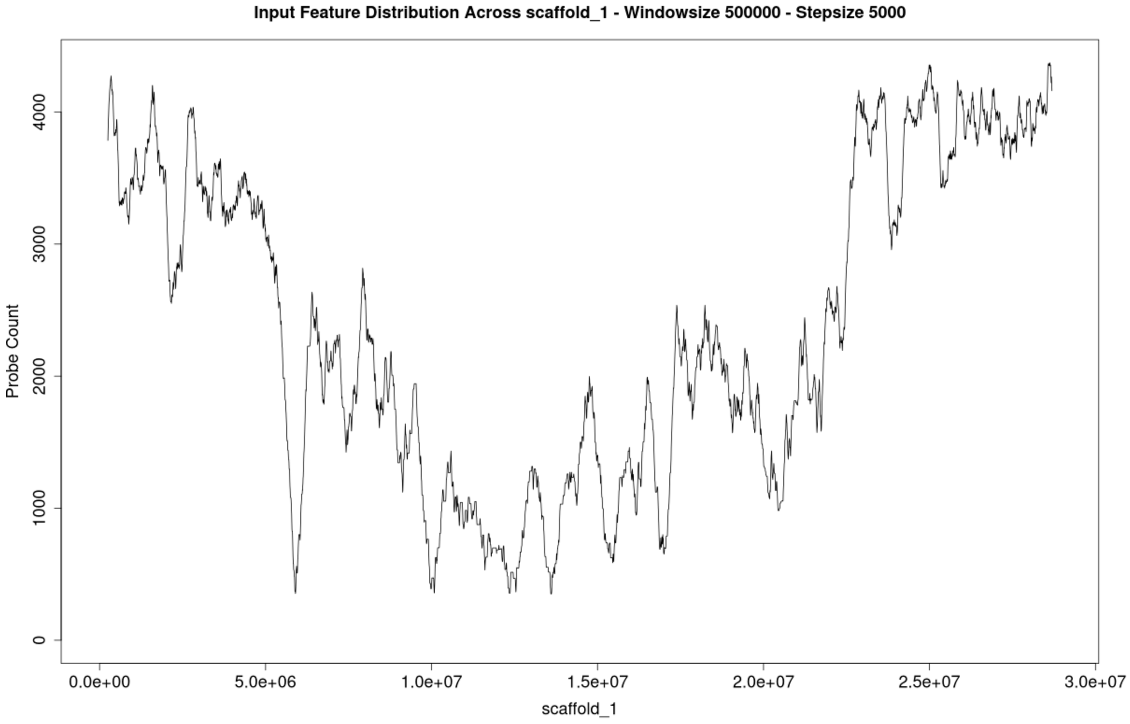

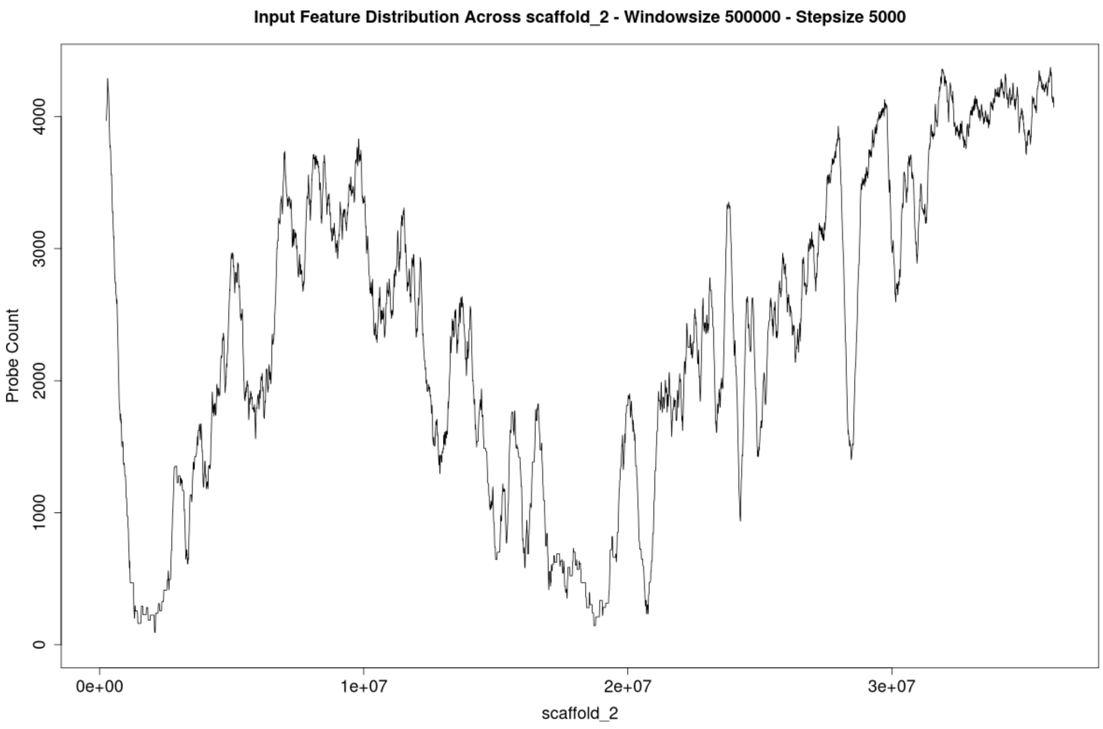
Input Feature Distribution Across scaffold_2 - Windowsize 500000 - Stepsize 5000
Probe Count
0
1000
2000
3000
4000
0e+00
1e+07
2e+07
3e+07
scaffold_2

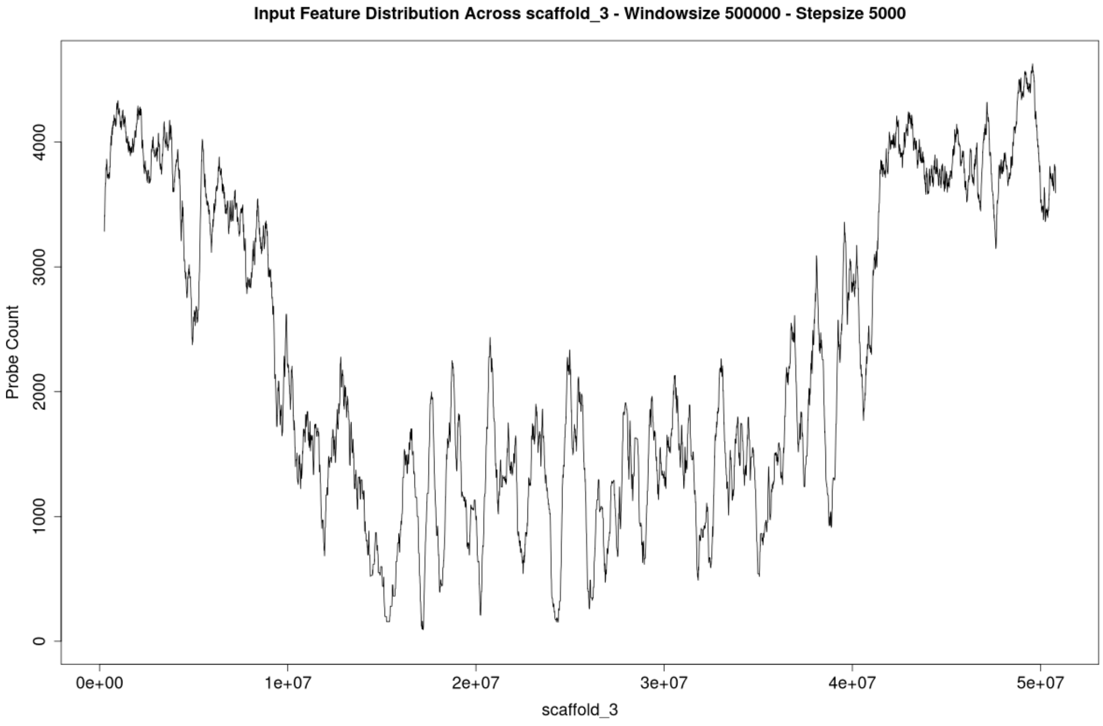
Input Feature Distribution Across scaffold_3 - Windowsize 500000 - Stepsize 5000
Probe Count
0
1000
2000
3000
4000
0e+00
1e+07
2e+07
3e+07
4e+07
5e+07
scaffold_3

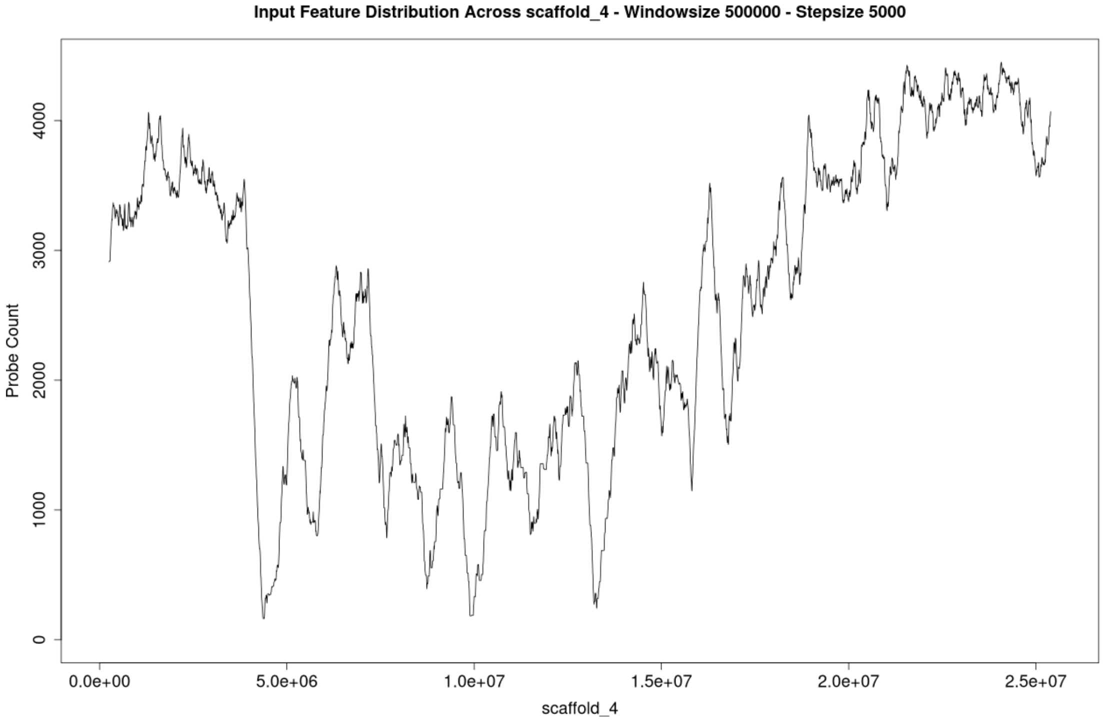
Input Feature Distribution Across scaffold_4 - Windowsize 500000 - Stepsize 5000
Probe Count
0
1000
2000
3000
4000
0.0e+00
5.0e+06
1.0e+07
1.5e+07
2.0e+07
2.5e+07
scaffold_4

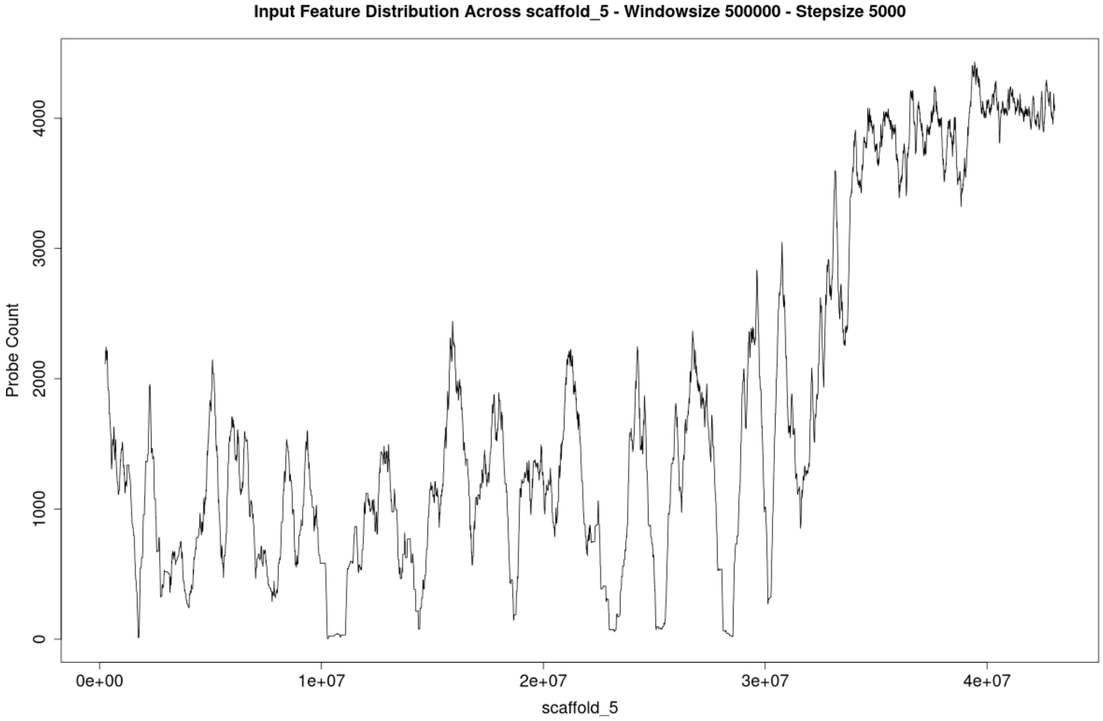
Input Feature Distribution Across scaffold_5 - Windowsize 500000 - Stepsize 5000
Probe Count
0
1000
2000
3000
4000
0e+00
1e+07
2e+07
3e+07
4e+07
scaffold_5

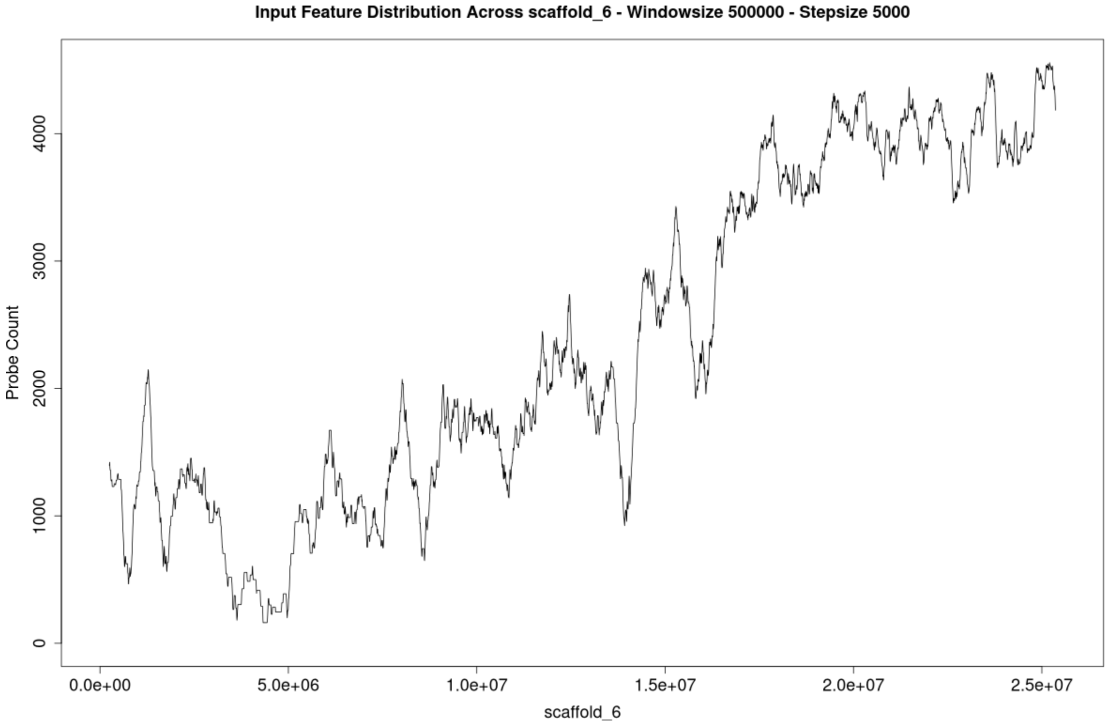

Input Feature Distribution Across scaffold_6 - Windowsize 500000 - Stepsize 5000
Probe Count
0
1000
2000
3000
4000
0.0e+00
5.0e+06
1.0e+07
1.5e+07
2.0e+07
2.5e+07
scaffold_6

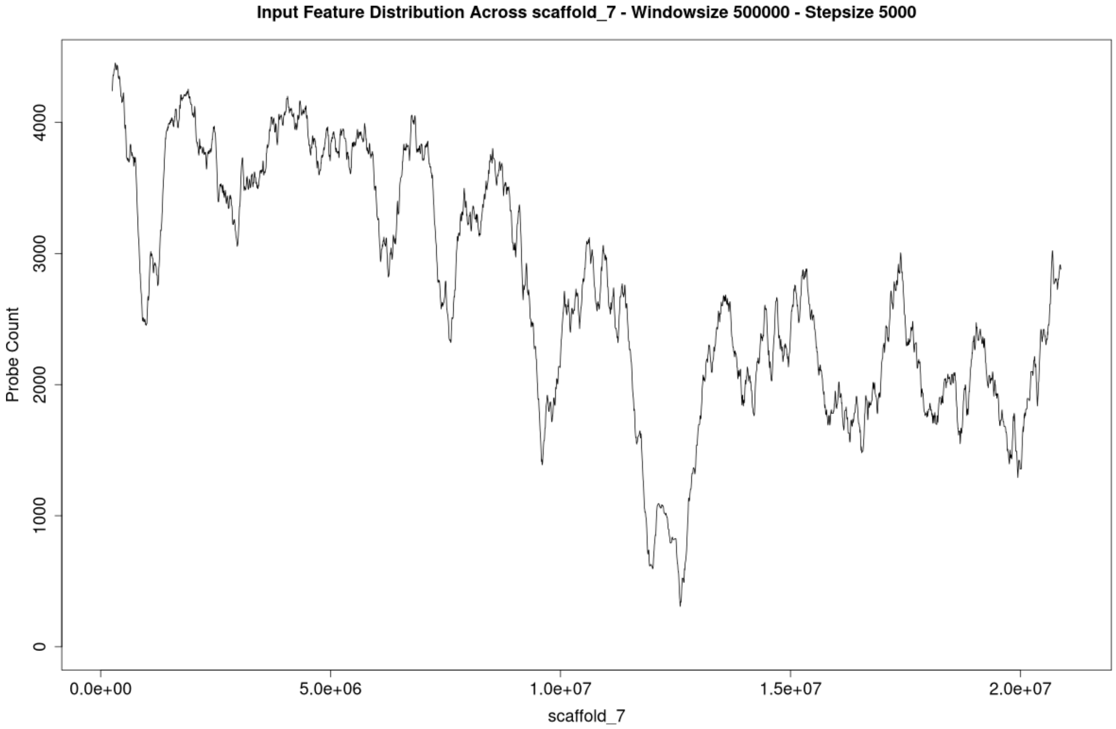

Input Feature Distribution Across scaffold_7 - Windowsize 500000 - Stepsize 5000
Probe Count
0
1000
2000
3000
4000
0.0e+00
5.0e+06
1.0e+07
1.5e+07
2.0e+07
scaffold_7

Input Feature Distribution Across scaffold_8 - Windowsize 500000 - Stepsize 5000

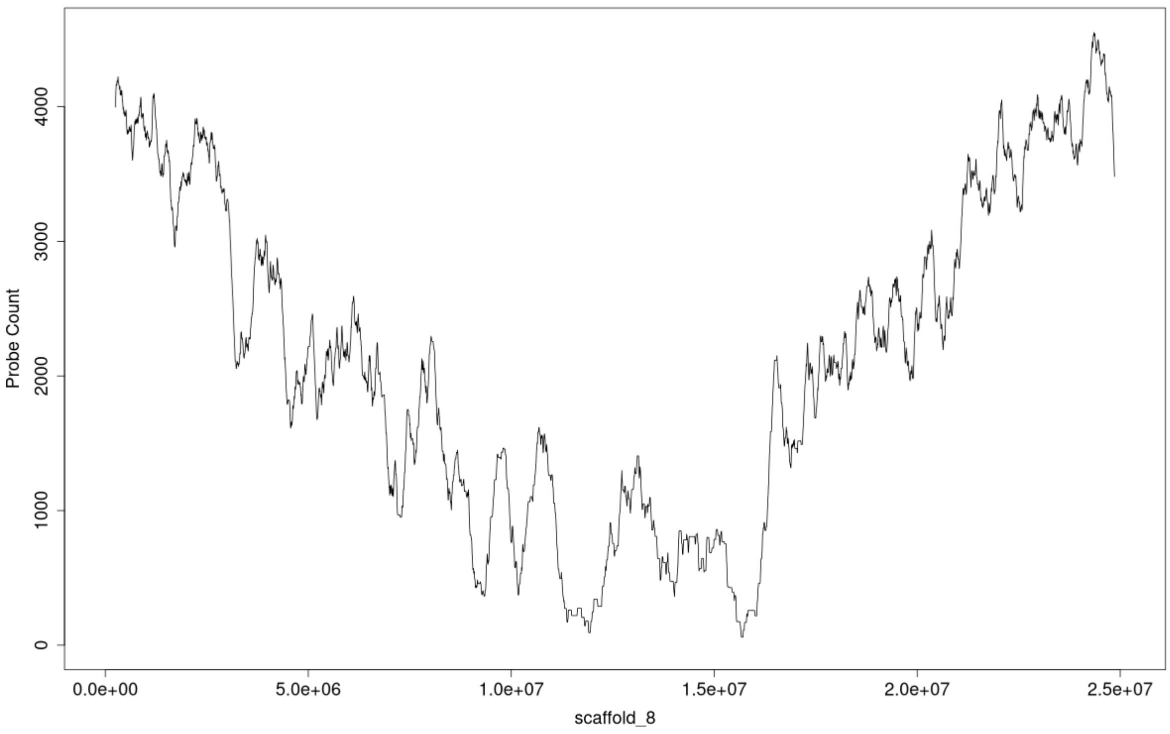

Input Feature Distribution Across scaffold_9 - Windowsize 500000 - Stepsize 5000

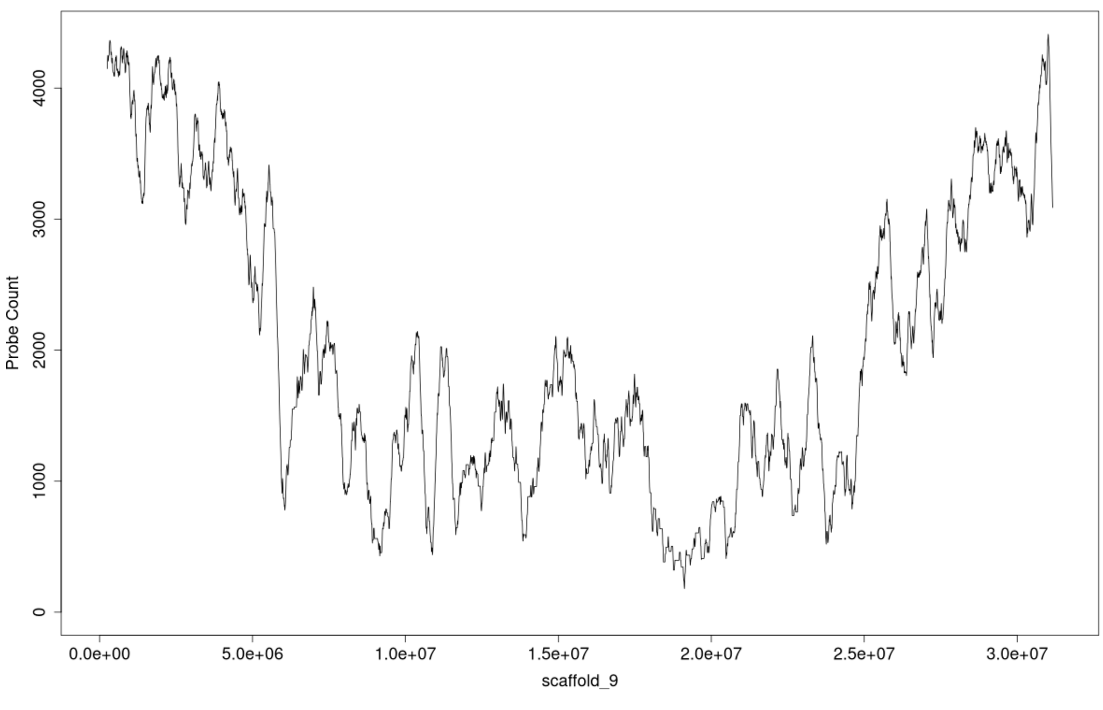

Figure S3 Venn diagram of 57,933 variants for the Axiom® Citrus Genotyping Array. Venn diagram showing the number of polymorphic variants, after variant filtering part 1, contributed by each group represented in the discovery panel and used on the Axiom® Citrus Genotyping Array.

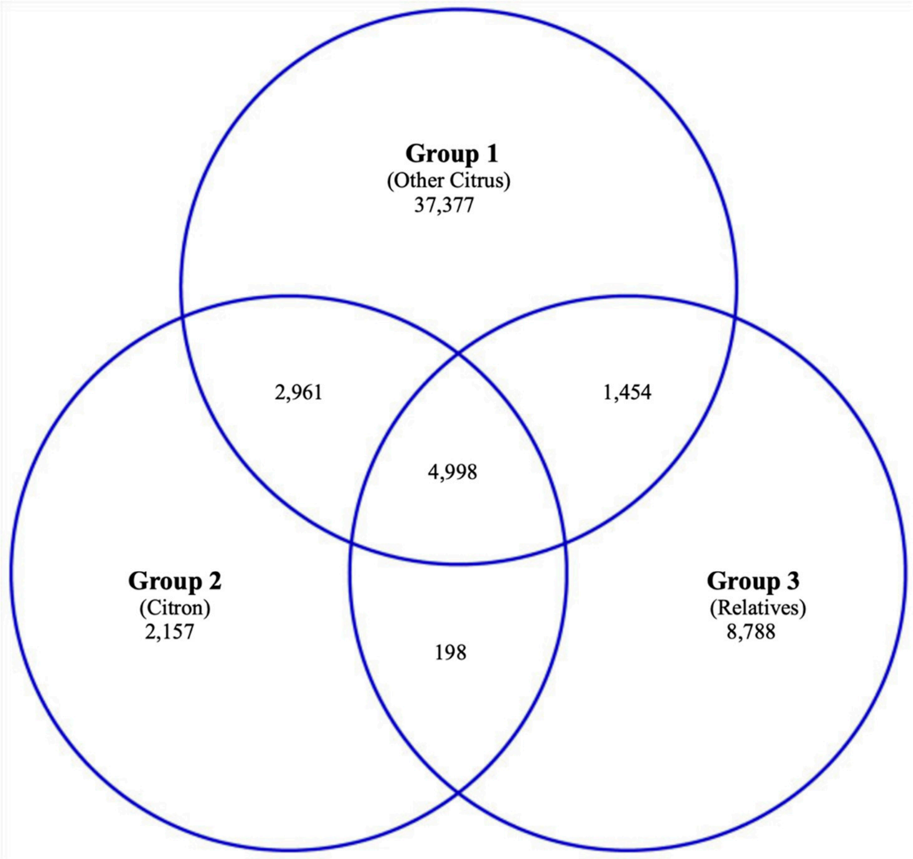

Figure S4 Distribution of SNPs represented on the Axiom® Citrus Genotyping Array on each of nine chromosomes. SNP distribution is plotted against the Phytozome *C. clementina v1.0* reference genome on chromosomes 1-9 (a-i) with 500kb window size and 5kb stepsize

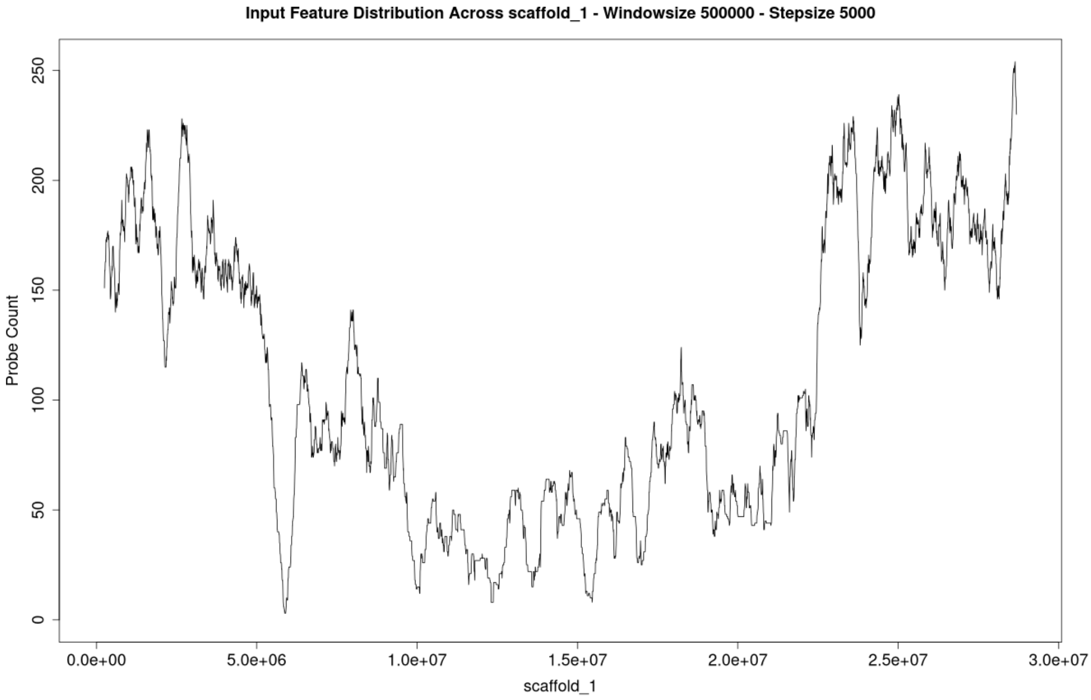

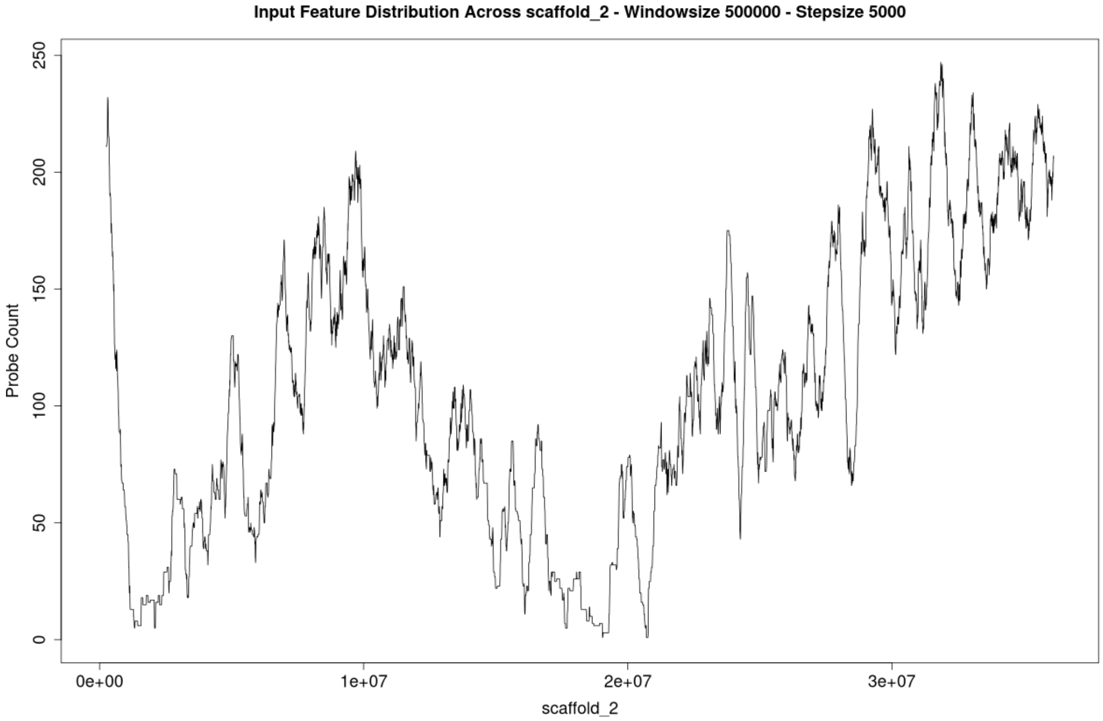
Input Feature Distribution Across scaffold_2 - Windowsize 500000 - Stepsize 5000
Probe Count
0
50
100
150
200
250
0e+00
1e+07
2e+07
3e+07
scaffold_2

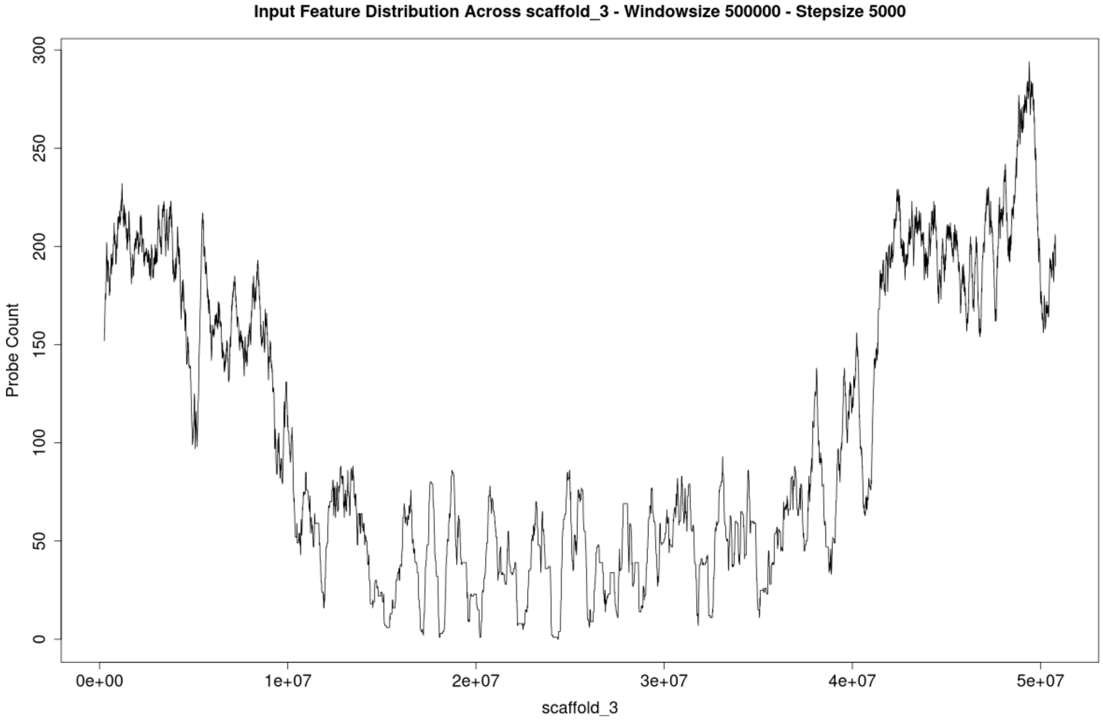
Input Feature Distribution Across scaffold_3 - Windowsize 500000 - Stepsize 5000
Probe Count
0
50
100
150
200
250
300
0e+00
1e+07
2e+07
3e+07
4e+07
5e+07
scaffold_3

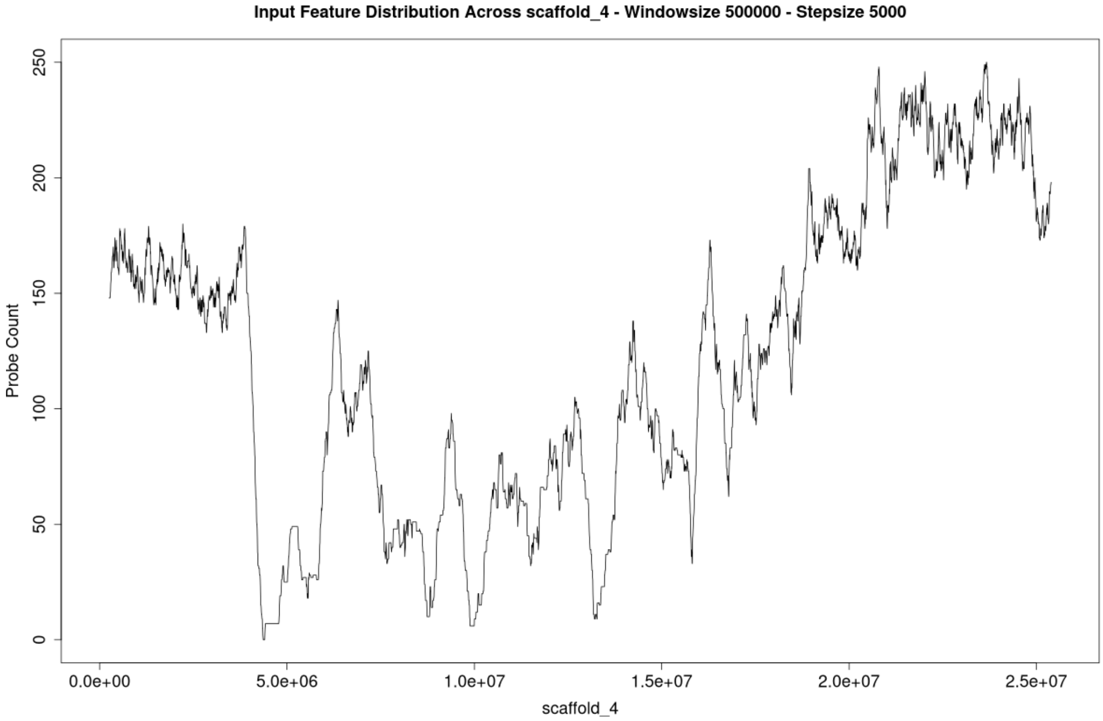
Input Feature Distribution Across scaffold_4 - Windowsize 500000 - Stepsize 5000
Probe Count
0
50
100
150
200
250
0.0e+00
5.0e+06
1.0e+07
1.5e+07
2.0e+07
2.5e+07
scaffold_4

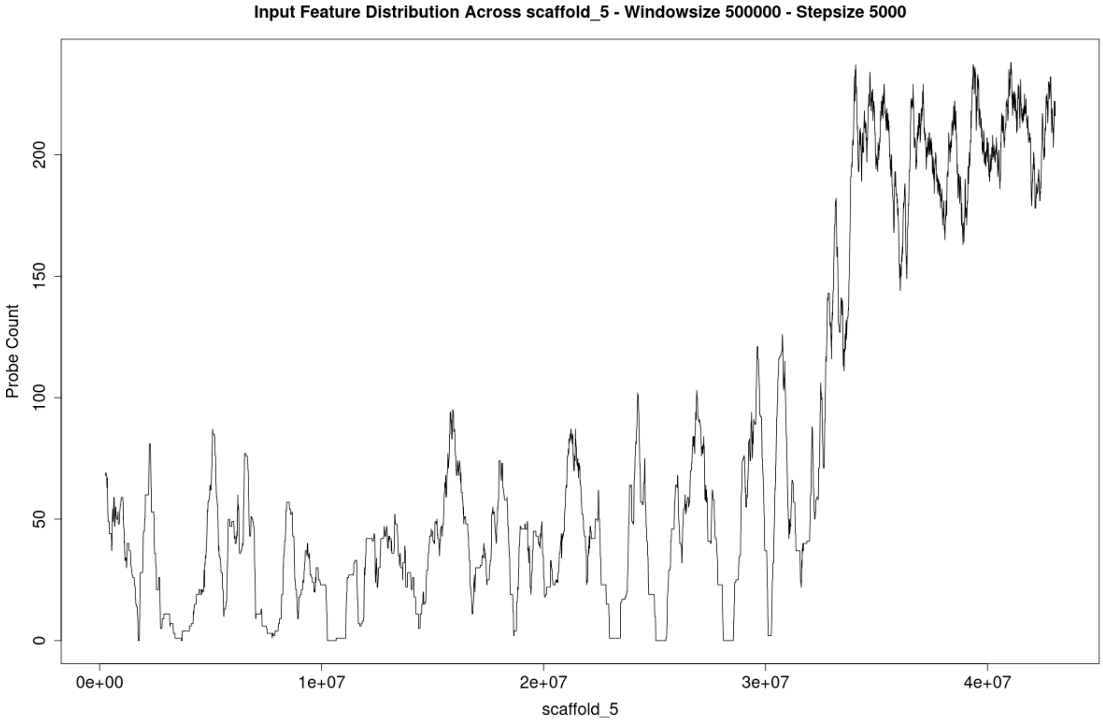
Input Feature Distribution Across scaffold_5 - Windowsize 500000 - Stepsize 5000
Probe Count
0
50
100
150
200
0e+00
1e+07
2e+07
3e+07
4e+07
scaffold_5

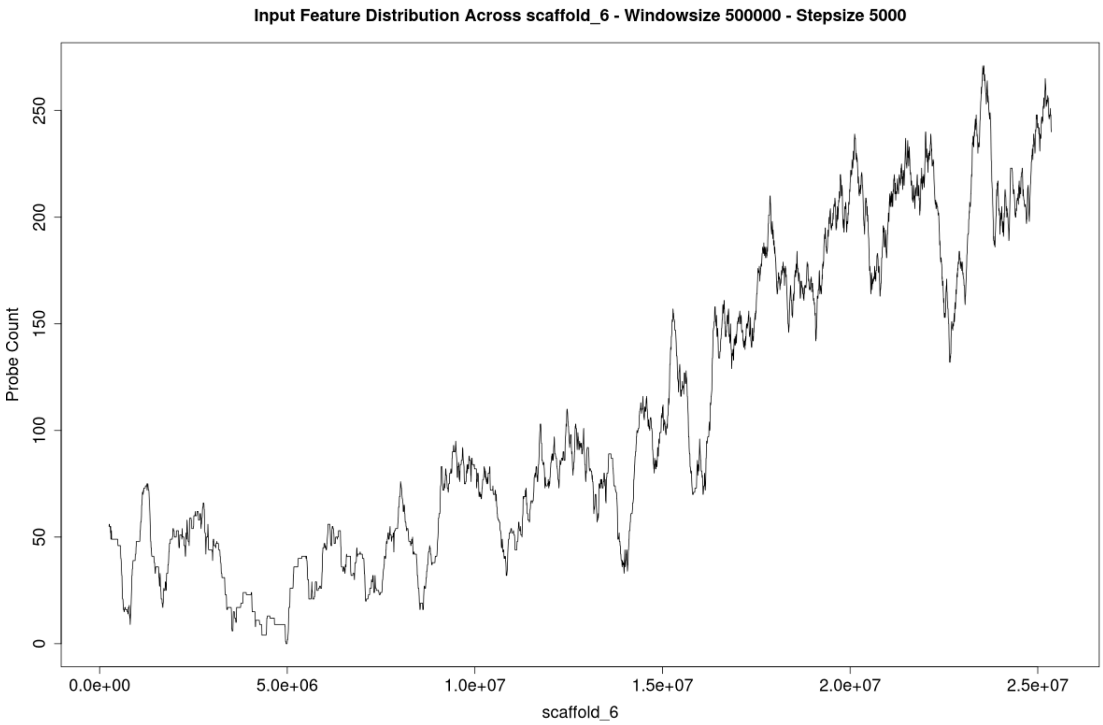
Input Feature Distribution Across scaffold_6 - Windowsize 500000 - Stepsize 5000
Probe Count
0
50
100
150
200
250
0.0e+00
5.0e+06
1.0e+07
1.5e+07
2.0e+07
2.5e+07
scaffold_6

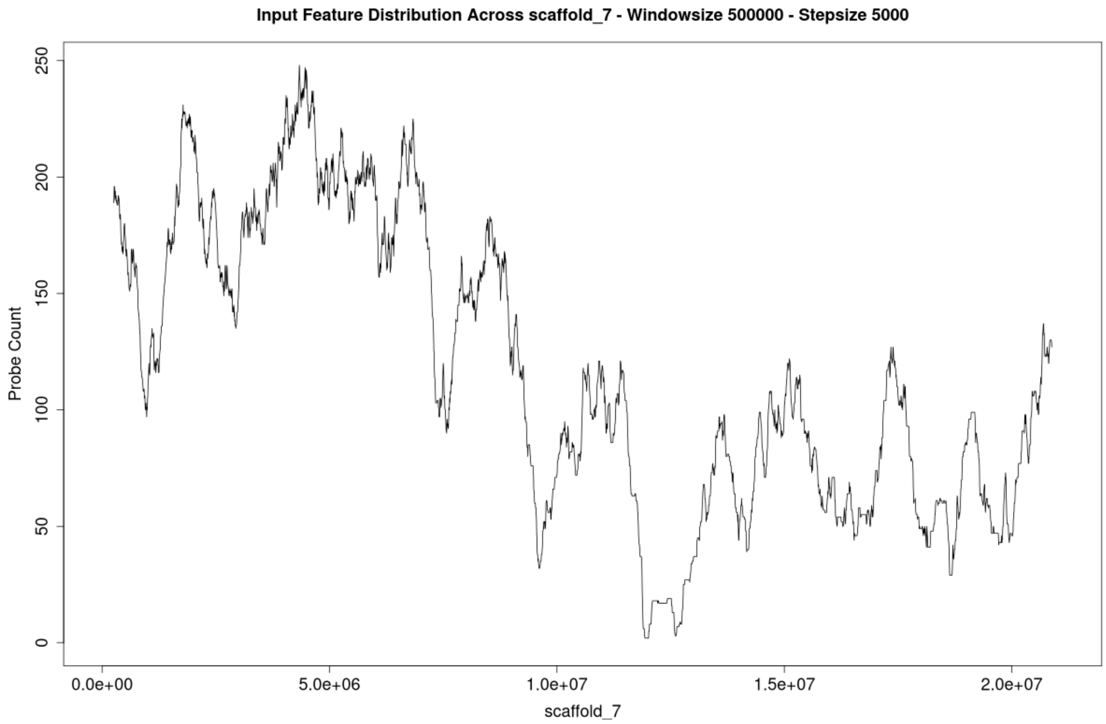
Input Feature Distribution Across scaffold_7 - Windowsize 500000 - Stepsize 5000
Probe Count
0
50
100
150
200
250
0.0e+00
5.0e+06
1.0e+07
1.5e+07
2.0e+07
scaffold_7

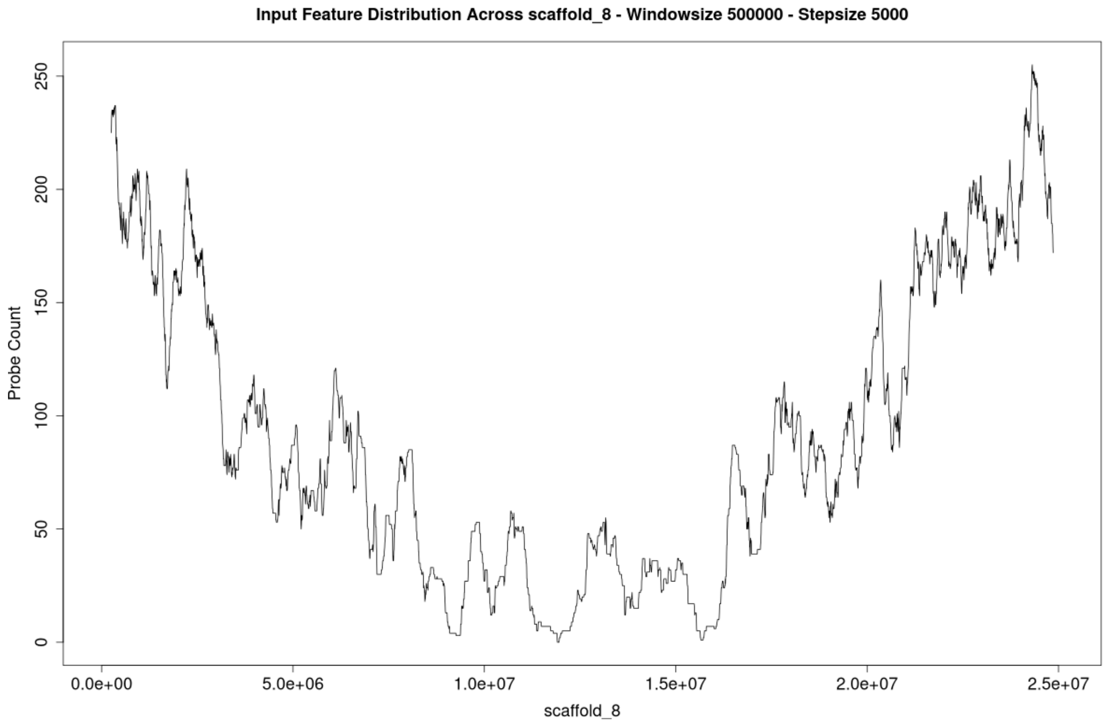
Input Feature Distribution Across scaffold_8 - Windowsize 500000 - Stepsize 5000
Probe Count
0
50
100
150
200
250
0.0e+00
5.0e+06
1.0e+07
1.5e+07
2.0e+07
2.5e+07
scaffold_8

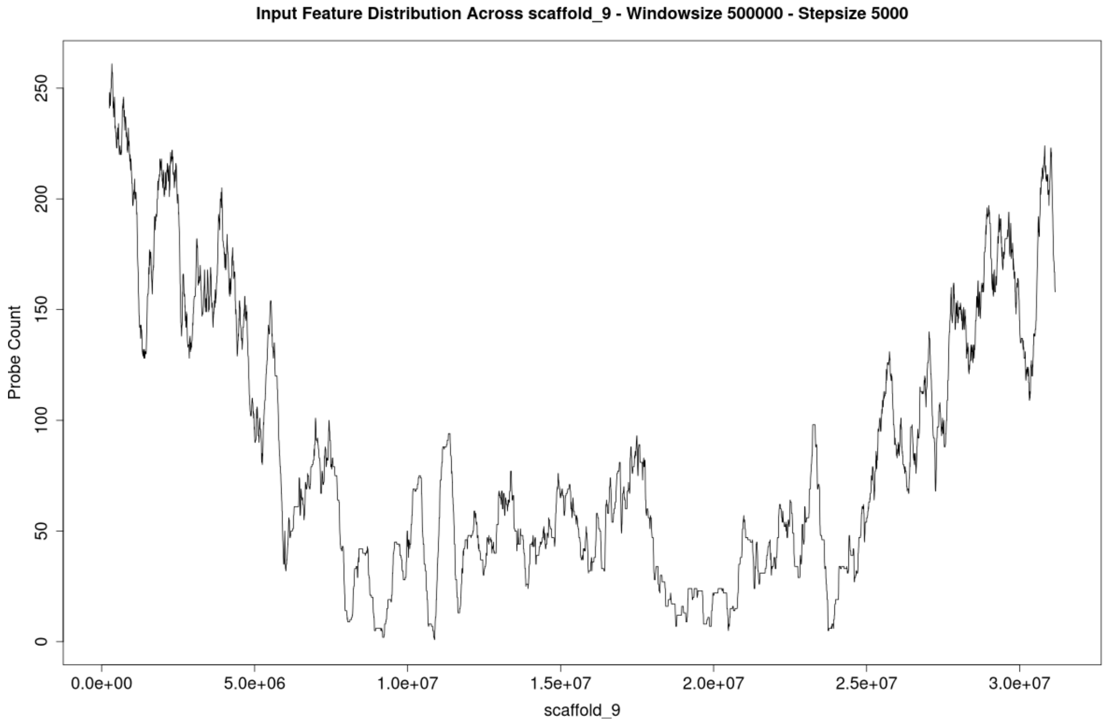
Input Feature Distribution Across scaffold_9 - Windowsize 500000 - Stepsize 5000
Probe Count
0
50
100
150
200
250
0.0e+00
5.0e+06
1.0e+07
1.5e+07
2.0e+07
2.5e+07
3.0e+07
scaffold_9

Figure S5 Topological comparison of two trees generated with data from the Axiom® Citrus Genotyping Array and sequence data of 26 accessions in the citrus variant discovery panel. Phylogenetic trees with the same 26 accessions and with different topologies were compared side by side with Phylo.io. Branch color scheme of yellow (0) to blue (1) indicates the similarities between the two trees. a) Tree generated by the Axiom® Citrus Genotyping Array data based on 41,626 concatenated SNPs. (b) Tree generated by sequence data with 32,754 SNPs.

a) b)

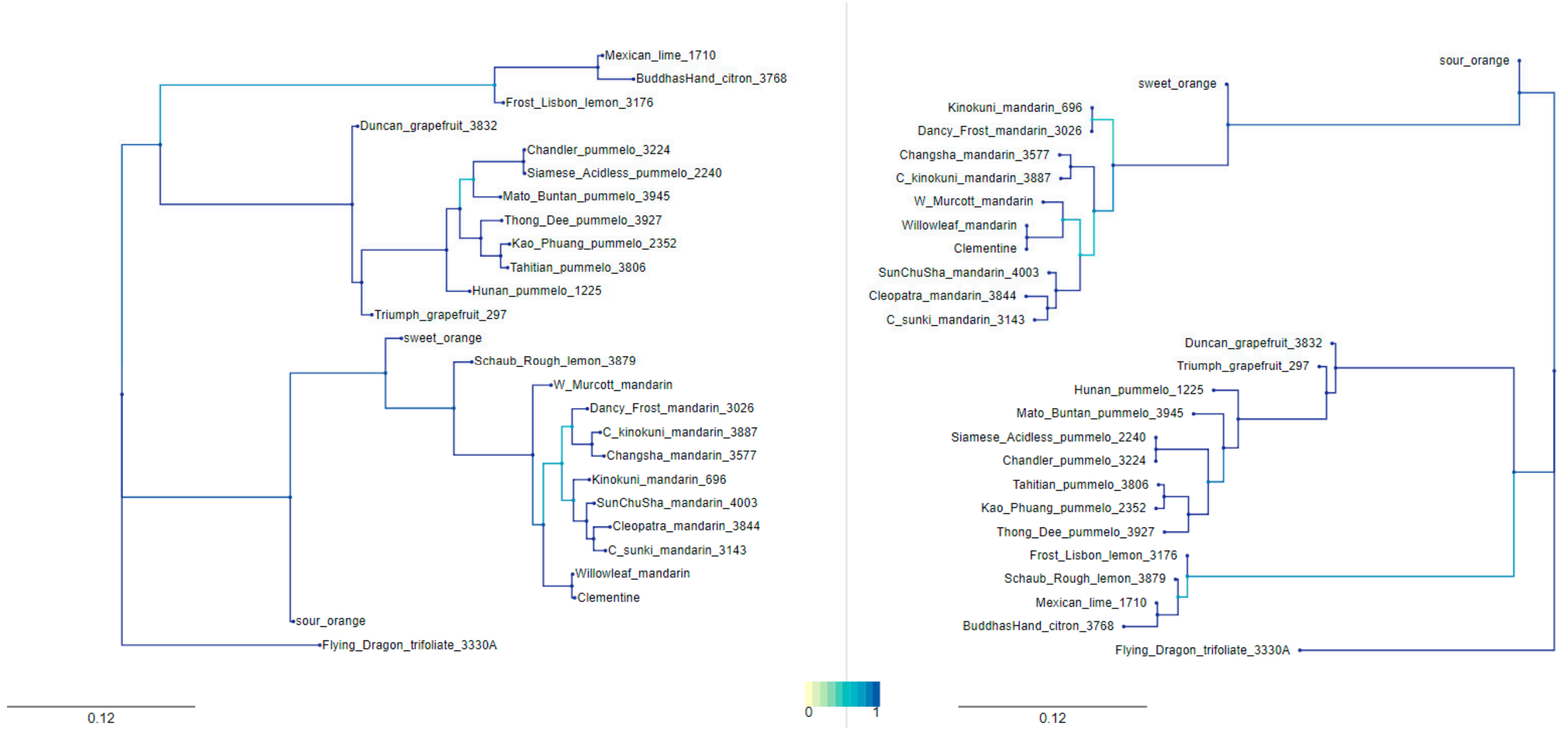

Figure S6. Structural annotation of markers represented on the Axiom® Citrus HD Genotyping Array (a) Percent distribution of the Axiom® Citrus HD Genotyping Array markers in gene body versus intergenic regions against Phytozome *C. clementina v1.0.* (b) Percent distribution of the Axiom® Citrus HD Genotyping Array markers in genome annotation against Phytozome *C. clementina v1.0.*

(a)

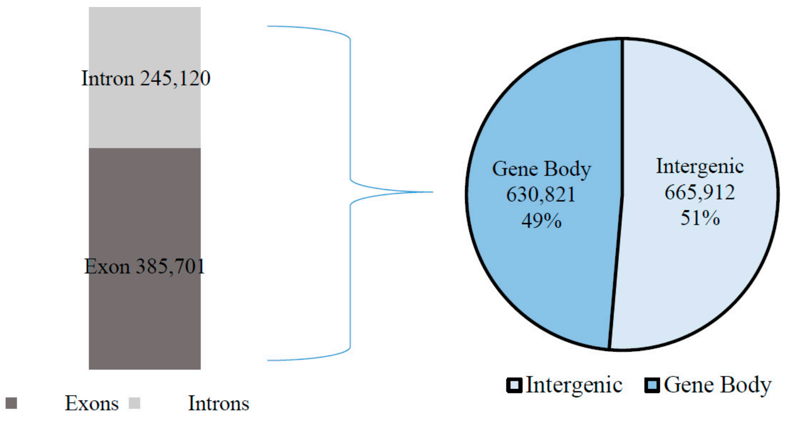

(b)

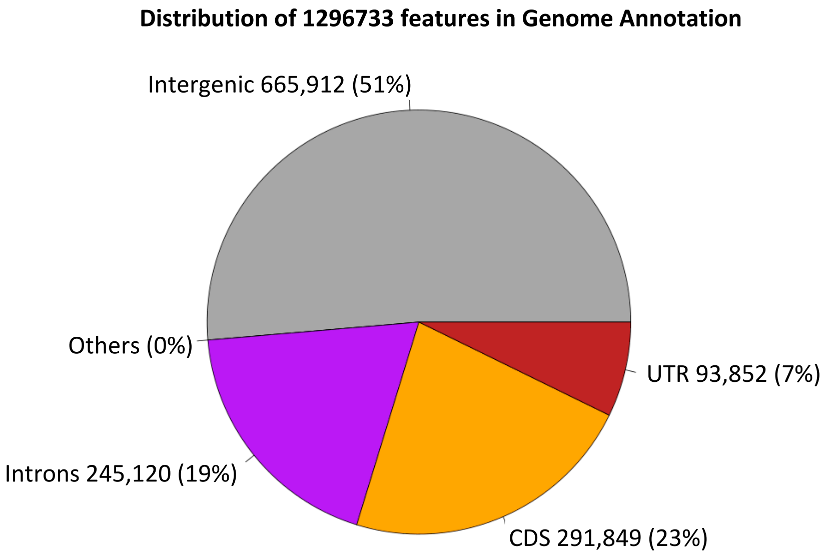

Figure S7 Structural annotation of markers represented on the Axiom® Citrus Genotyping Array (a) Percent distribution of the Axiom® Citrus Genotyping Array markers in gene body versus intergenic regions against Phytozome *C. clementina v1.0.* (b) Percent distribution of the Axiom® Citrus Genotyping Array markers in genome annotation against Phytozome *C. clementina v1.0.*

(a)

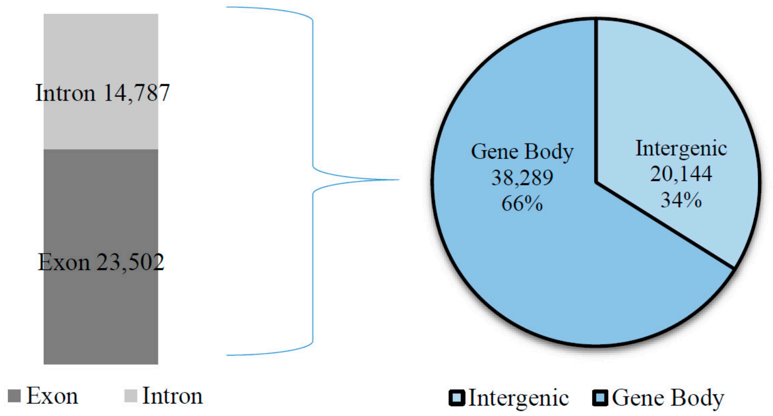

(b)

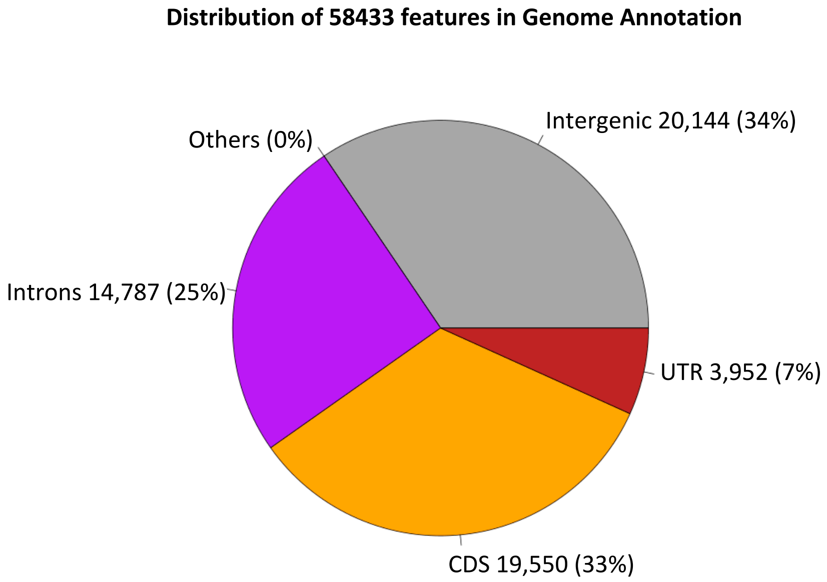

Figure S8. Genotyping error simulation of 26 CVC accessions including 25 accessions in citrus variant discovery panel and Carrizo citrange CRC 2863.

26 CVC accessions included 1 citron (orange), 9 mandarins (yellow), 6 pummelos (green), 1 trifoliate (blue), 1 trifoliate hybrid (dark blue), 1 clementine (gray), 2 grapefruits (pink), 1 lemon (brown), 1 lime (light blue), 1 rough lemon (red), 1 sour orange (purple) and 1 sweet orange (black). PCA was performed with 14,470 loci of the Axiom® Citrus Genotyping Array and PC1, PC2, and PC3 in the data was compared. Both PC1 (43.7 %) and PC2 (25 %) showed no differences for 0%, 3% and 5 % error. PC3 was 14.9 %, 14.7 % and 14.8 % for 0%, 3% and 5 % error, respectively. a) PCA with no genotyping call errors. b) PCA with 3 % genotyping error in Flying Dragon CRC 3330A and Carrizo CRC 2863. c) PCA with 5 % genotyping error in Flying Dragon CRC 3330A and Carrizo CRC 2863.

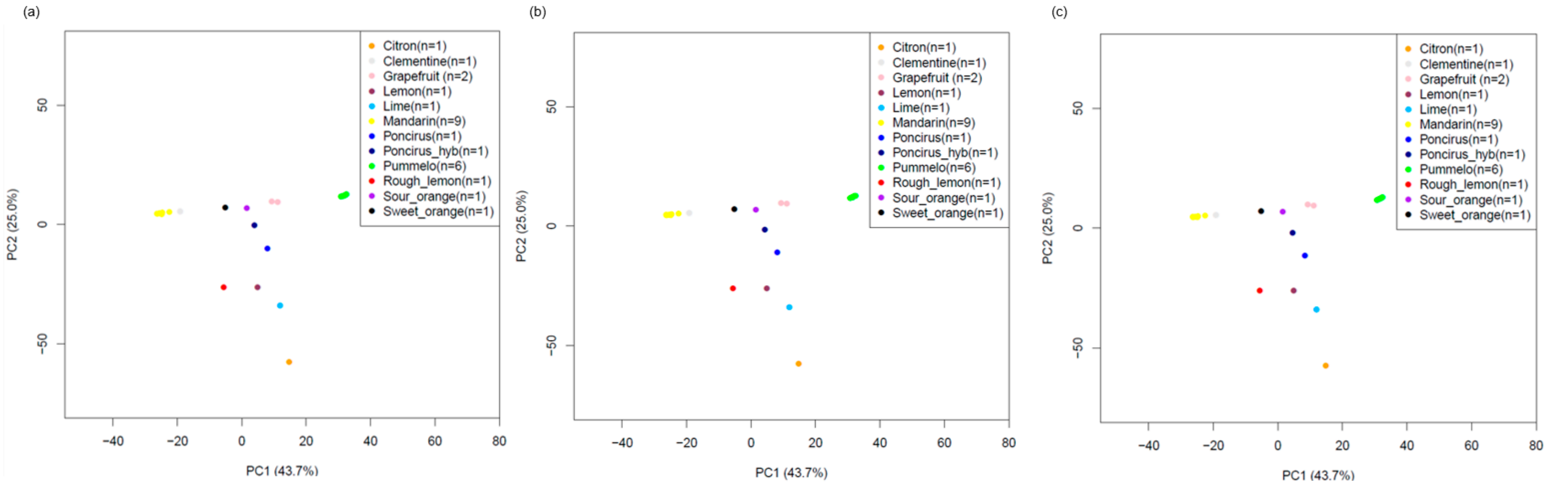

Figure S9. Effect of genotyping error on phylogenetic trees of 26 CVC accessions including 25 accessions in the citrus variant discovery panel and Carrizo citrange CRC 2863. Neighbor-joining trees were constructed with 14,470 loci of the Axiom® Citrus Genotyping Array and pairwise tree statistical comparison was performed. Carrizo citrange 2863 was not represented in the discovery panel, therefore, haplotype data inferred from whole genome amplified single pollen grains genotyped with the Axiom® Citrus Genotyping Array was used as a substitute for the sequence data. Topological differences among trees by PH85 were 0.003595658, 0.002188715, and 0.001411884 for 0 % vs 5 % error, 0 % vs 3% error, and 3 % vs 5% error, respectively and found statistically not significant. a) Neighbor-joining tree with no miss-matched genotyping call against sequence data and haplotype data. b) Neighbor-joining tree with 3 % genotyping error in Flying Dragon CRC 3330A and Carrizo CRC 2863 against sequence data and haplotype data. c) Neighbor-joining tree with 5 % genotyping error in Flying Dragon CRC 3330A and Carrizo CRC 2863 against sequence data and haplotype data.

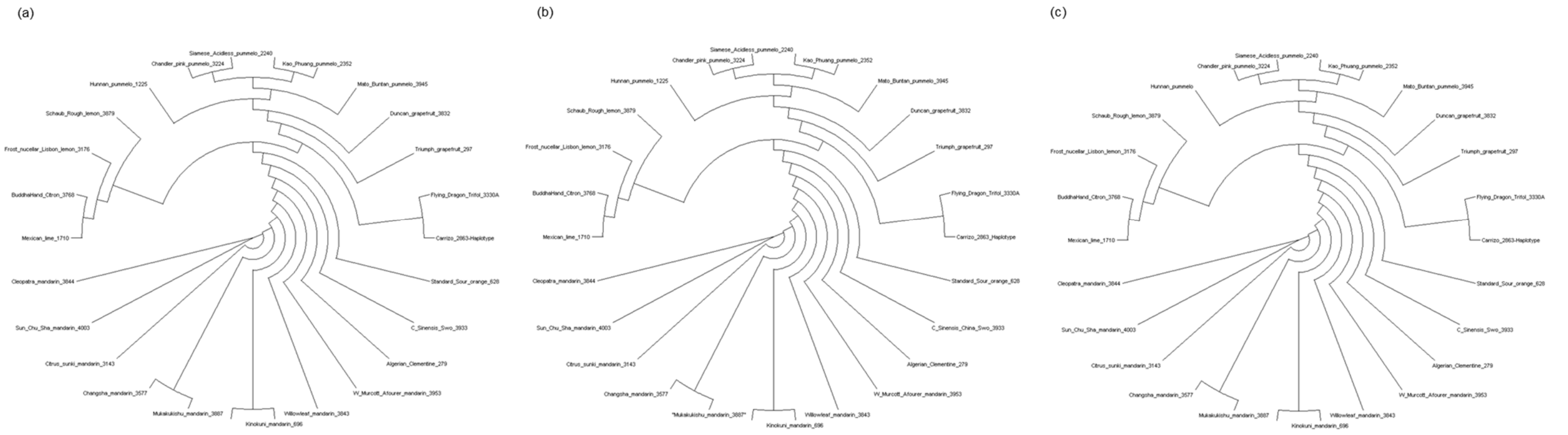

Figure S10. Effect of simulated genotyping errors on Admixture analysis of 26 CVC accessions including 25 accessions in the citrus variant discovery panel and Carrizo citrange CRC 2863. ADMIXTURE was performed with 14,470 loci of the Axiom® Citrus Genotyping Array. 10-fold cross validation was used for log-likelihood to determine the appropriate number of K in the data. Due to exclusion of some taxa from the analyses, some accessions appear to be admixed with taxa most closely related to the actual donor. There was no difference in the results for 0%, 3% and 5 % error in Flying Dragon and Carrizo at K=4. a) Admixture plot with 0% genotyping call errors at K=4. b) Admixture plot with 3% genotyping call errors at K=4. c) Admixture plot with 5% genotyping call errors at K=4.

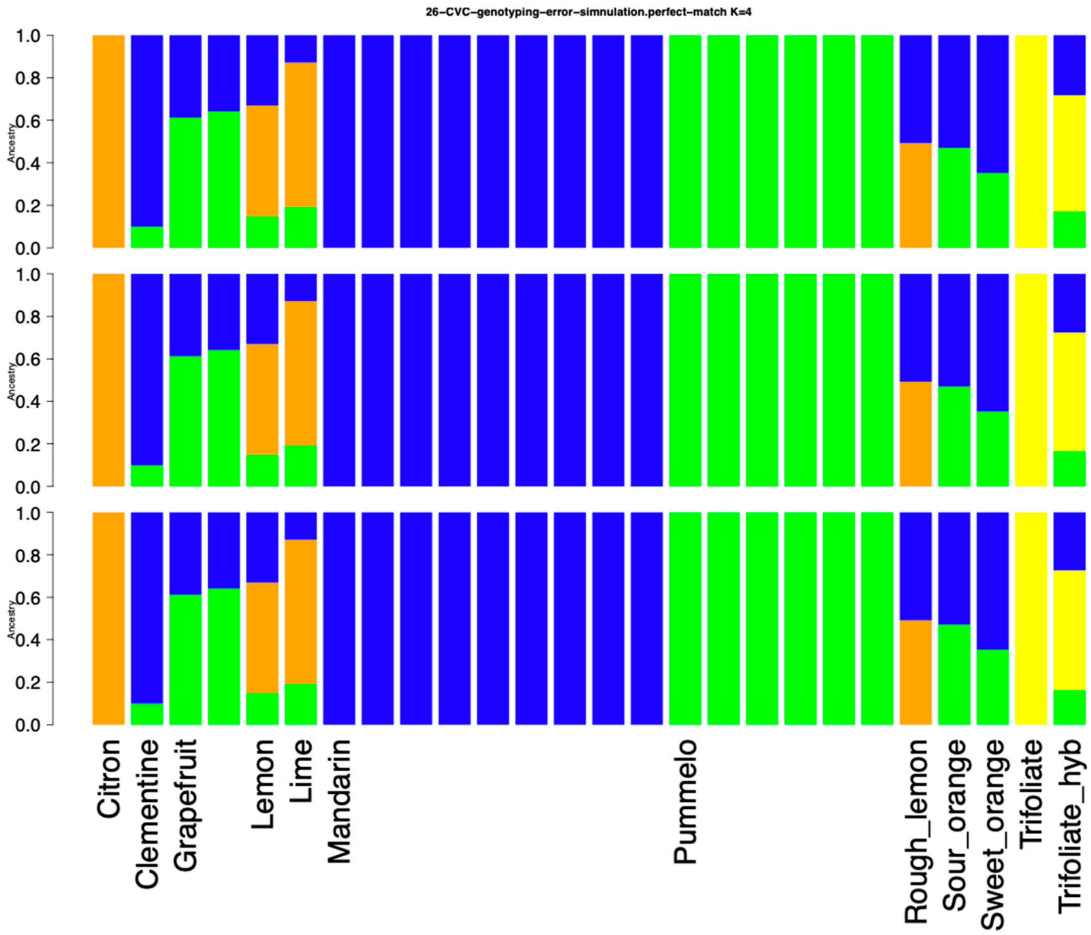

Figure S11. Neighbor joining phylogeny tree generated with sequence data based on 2,251,517 concatenated SNPs. The 29 accessions considered for the analysis included mostly non-hybrid and non-admixed accessions in the variant discovery panel. Some admixed mandarins were included because non-admixed mandarins were not sequenced. 2,251,517 SNPs were generated with SNPhylo with filter setting 1 for LD, 0.0 for MAF, and 1 for missing value. Neighbor-oining tree was generated with TASSEL v5.2. and visualized with dendroscope 3 (http://dendroscope.org/).

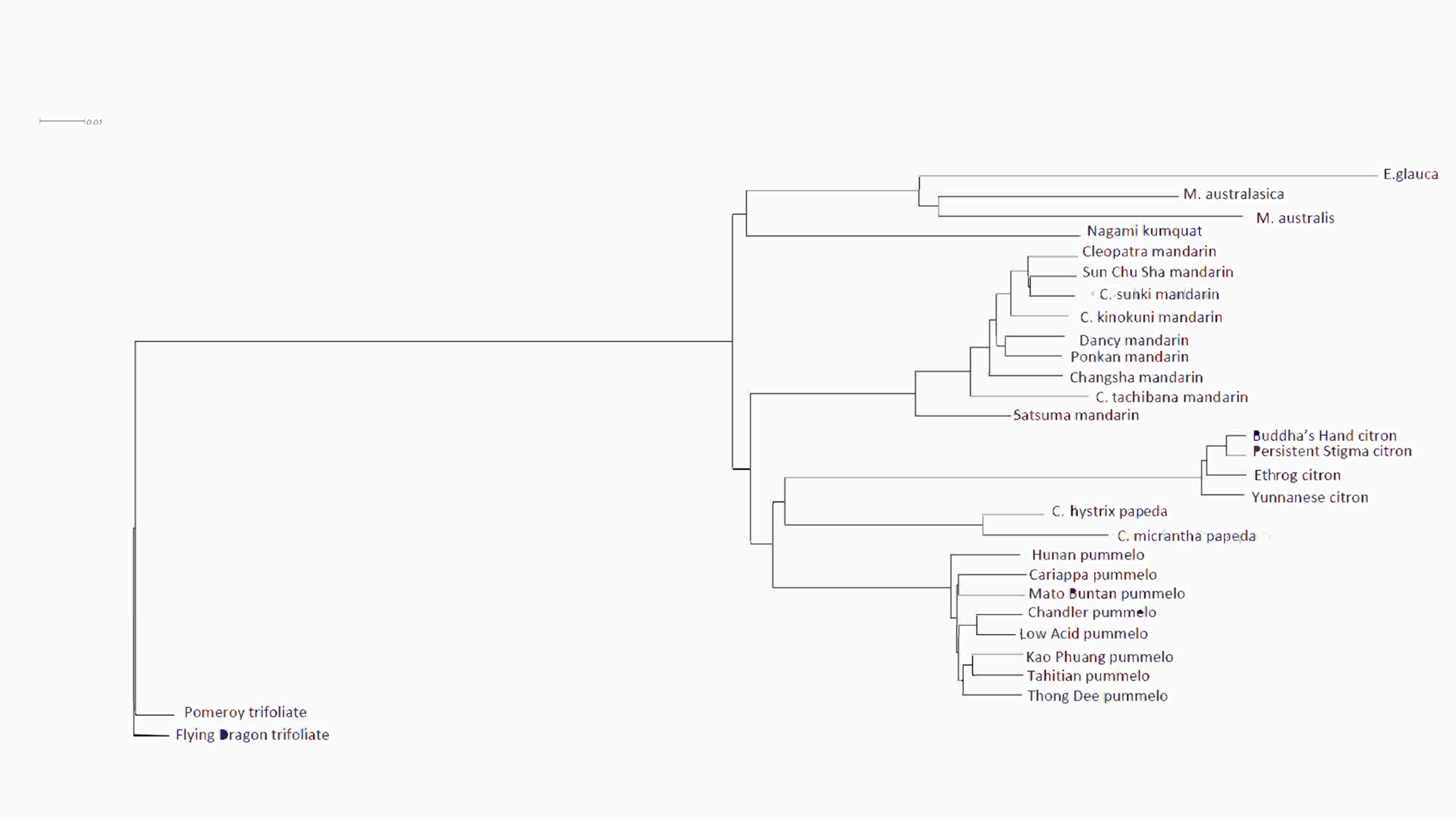

31

Figure S12 Venn diagram of 5.3 M (SNPs and INDELs) variants of initial candidate marker loci for designing the Axiom® Citrus HD Genotyping Array. Venn diagram shows the number of polymorphic variants in each group of the variant discovery panel, after variant filtering part 1, submitted to the Affymetrix bioinformatics team for in silico analysis of the variants to predict conversion performance in the array.

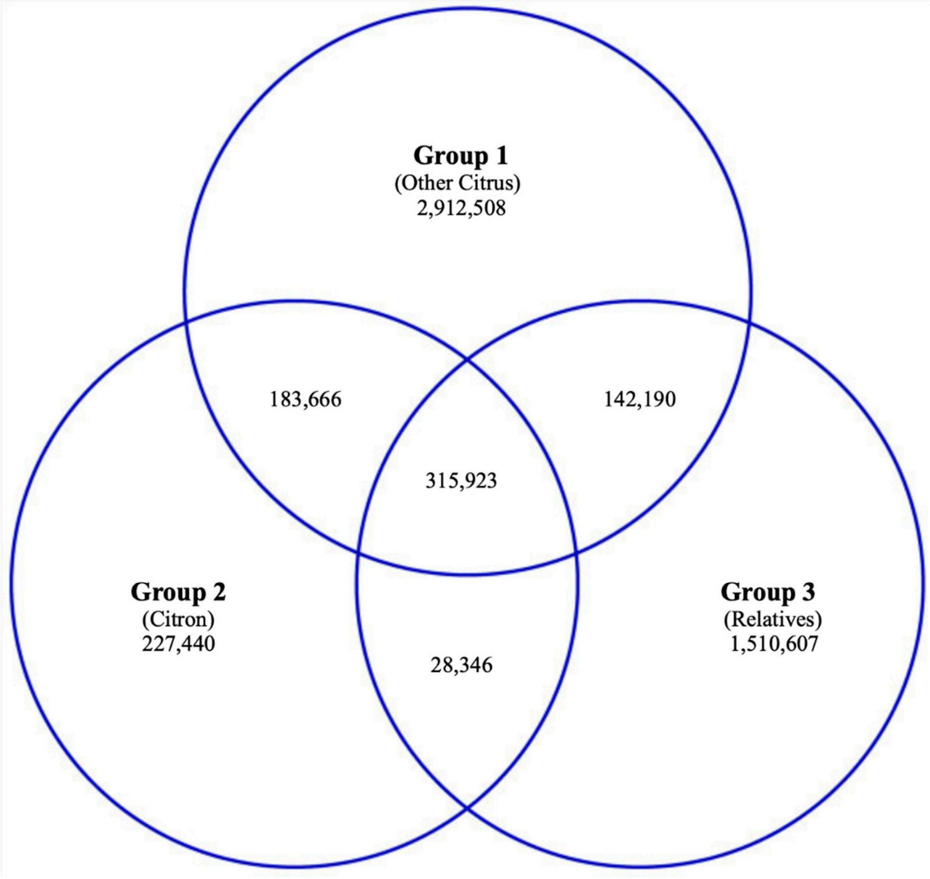

Figure S13 Venn diagram of variant sites used as QC probes (20,000) for the Axiom® Citrus HD Genotyping Array. Venn diagram shows the number of polymorphic variants, after variant filtering part 1, contributed by each group represented in the discovery panel and used as QC probes on the Axiom® Citrus HD Genotyping Array.

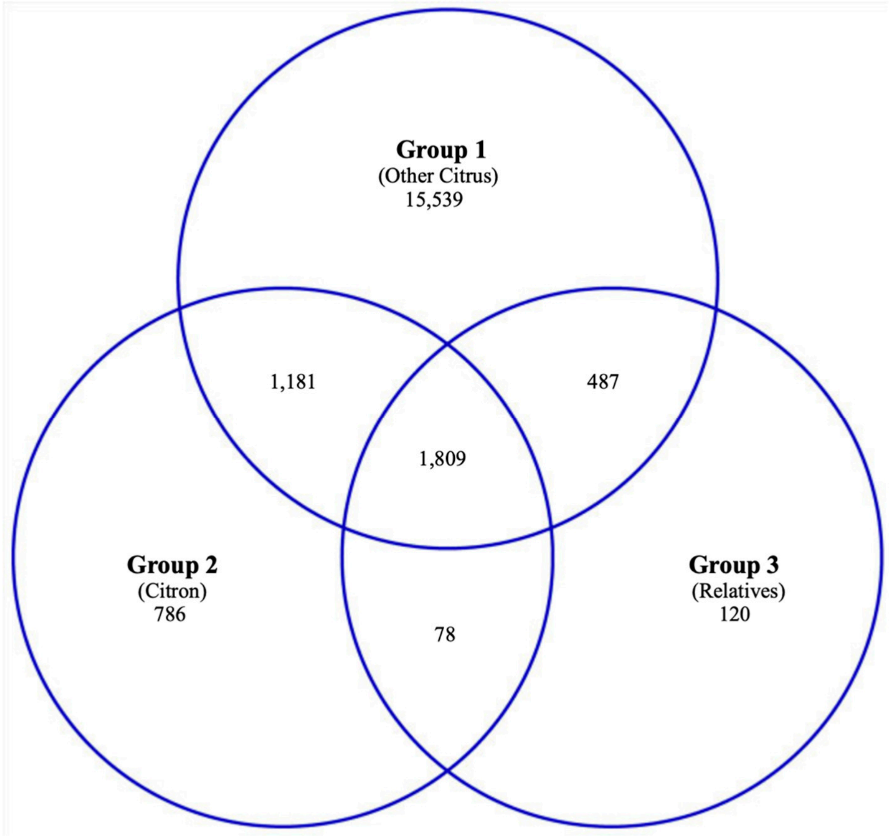

Supplement: Supplementary file 1 [file plants-13-00691-s001.zip › plants-2823247-supplementary-Figs 021124.pdf]
